# A novel laparoscopic assisted mediastinal dissection with gastric tube inversion technique for gastric tube cancer reconstructed through a retrosternal route

**DOI:** 10.1002/ags3.12473

**Published:** 2021-05-24

**Authors:** Tetsuya Abe, Yoshihisa Numata, Eiji Higaki, Takahiro Hosoi, Yasuhiro Shimizu

**Affiliations:** ^1^ Department of Gastroenterological Surgery Aichi Cancer Center Hospital Nagoya Japan

**Keywords:** gastric tube cancer, retrosternal reconstruction, video‐assisted surgery

## Abstract

A median sternotomy is often performed in patients with gastric tube cancer reconstructed through the retrosternal route; however, this procedure is invasive and has the risk of severe infectious complications. To overcome these problems, we created a novel method to perform the reconstructed gastric tube resection using a gastric tube inversion technique combined with a laparoscopic mediastinal approach. After the duodenum was divided, the oral side of the cut end was sutured with silken threads for traction. The gastric tube was dissected from the caudal side under a laparoscopic mediastinal approach, whereas the cervical esophagus was taped. After the adhesion between the middle side of the posterior sternum and the reconstructed gastric tube was dissected to the cervix, the gastric tube was inverted by guiding and pulling the thread toward the cervical side. Sharp dissection was facilitated between the inverted gastric tube and the surrounding organs under moderate traction and a favorable surgical view. We have performed this procedure and evaluated the short‐term outcomes in six cases. The laparoscopic mediastinal approach was completed without a median sternotomy in all six cases. Restorable intraoperative lung injury was observed in one case and no major vessel injuries were observed. The postoperative course was satisfactory with a 29.5‐day median length of hospital stay (range, 16‐60 days). The gastric tube inversion technique combined with the laparoscopic mediastinal approach for patients with retrosternal‐reconstructed gastric tube cancer was shown to be safe and less invasive and should be considered in resection of the reconstructed gastric tube.

## Graphical abstract

We created the novel gastric tube inversion technique combined with laparoscopic mediastinal approach in gastric tube resection for the gastric tube cancer reconstructed through the retrosternal route. This approach is less invasive and safe, thereby avoiding the risk of serious complication associated with median sternotomy. Moreover, the gastric tube inversion technique helped to secure a sufficient working space in a narrow operation field and allows for safe dissection under favorable surgical view.

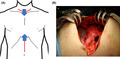

## 1 INTRODUCTION

With the advances in multidisciplinary treatment, the prognosis of esophageal cancer patients who have undergone esophagectomy has improved.^1^, ^2^ With the resulting increase in the long‐term survival of such patients, the incidence of gastric tube cancer has also increased.^3^, ^4^, ^5^

In patients with gastric tube cancer reconstructed through the retrosternal route, a median sternotomy is often performed^6^, ^7^, ^8^; however, this procedure is invasive and has the risk of severe complications, including sternal dehiscence or mediastinitis.^7^, ^9^

Recently, although there have been some reports of procedures without a median sternotomy for patients with retrosternal gastric tube cancer, nearly all of the reports only involve single cases.^7^, ^8^, ^9^, ^10^

Herein we report our novel gastric tube inversion technique combined with a laparoscopic mediastinal approach in performing radical total gastric tube resection for patients with gastric tube cancer reconstructed through the retrosternal route.

## 2 PATIENTS AND METHODS

### 2.1 Patients

We retrospectively reviewed medical records of patients who underwent total gastric tube resection for gastric tube cancer reconstructed through a retrosternal route at the Aichi Cancer Center Hospital between May 2009 and August 2020. This study was approved by the Review Board of Aichi Cancer Center Hospital (Approval No. ACC 2020‐1‐672). Regarding the surveillance after esophagectomy, patients received periodic physical and laboratory examinations at 3‐month intervals. Computed tomography was performed at 6‐month intervals, and endoscopic examination was performed annually in principle.

### 2.2 Surgical technique

**FIGURE 1:**
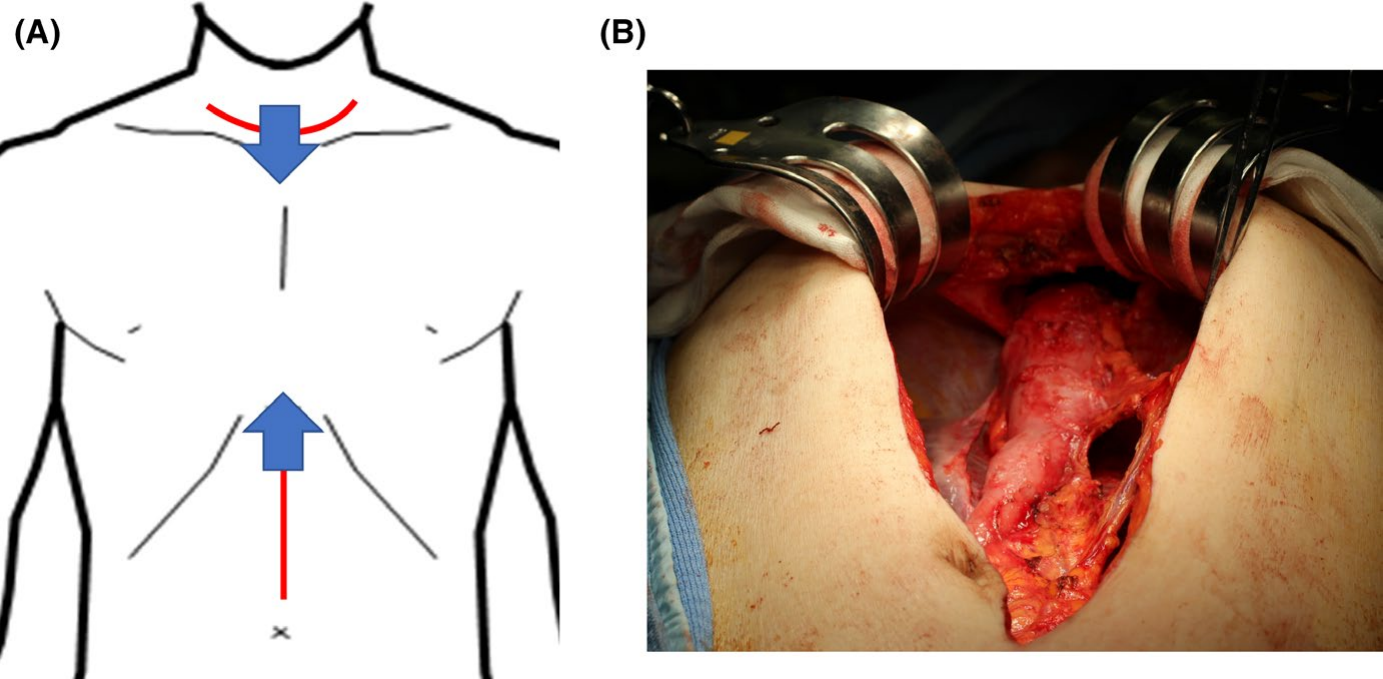
A, Schema of the skin incision. Arrows indicate direction of approach from the neck and abdominal incision. B, Intraoperative view using a Kent retractor. The clasps of a Kent retractor were hooked onto bilateral costal arches. The costal arches were lifted with an elevating handle to extend the entry of retrosternal space

**FIGURE 2:**
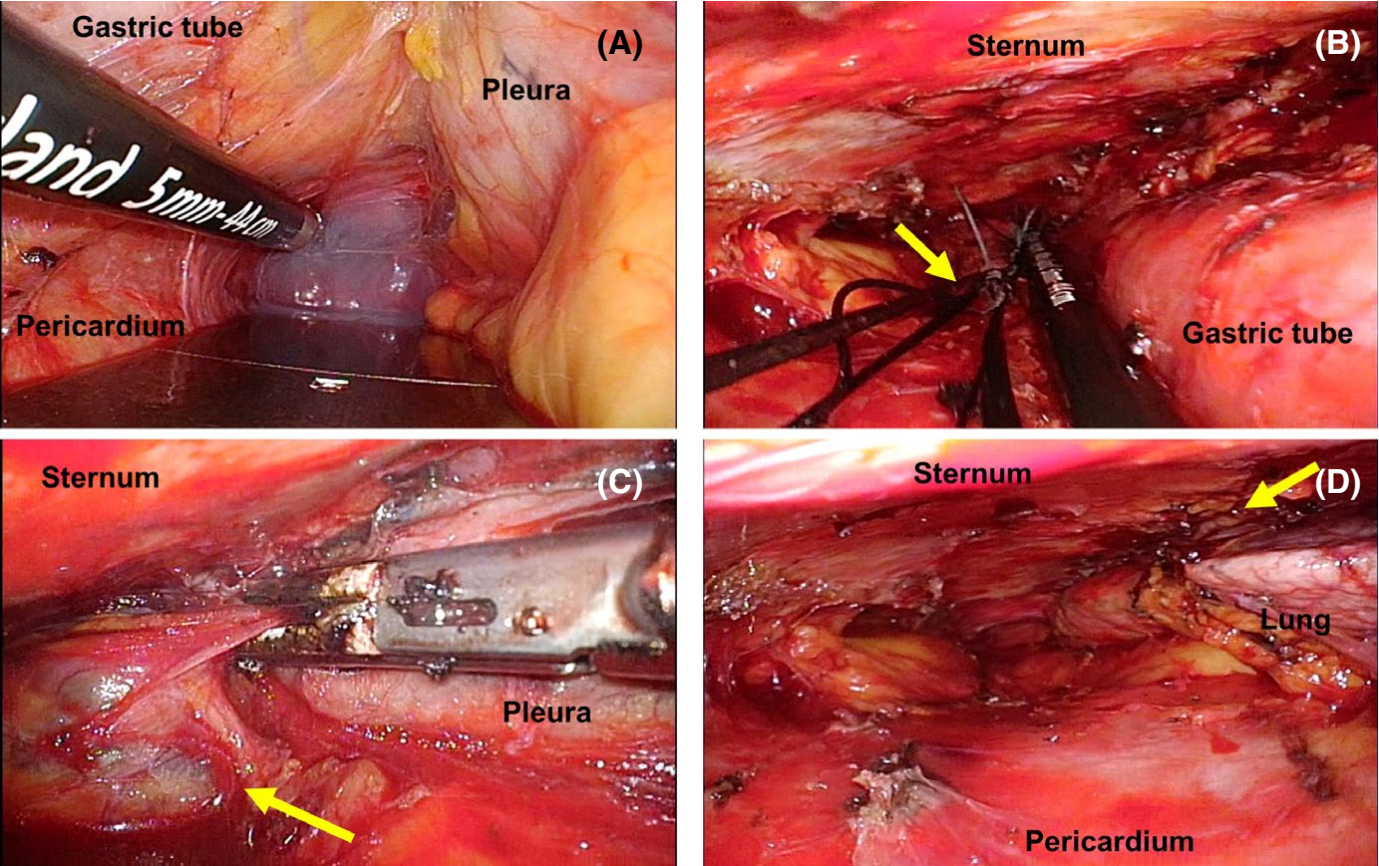
A, Dissection of the posterior adhesions. Posterior adhesions were relatively loose and bluntly dissected. B, Guiding the silken threads to the cervix. The threads sutured to the gastric side of the stump (yellow arrow) were guided to the cervix through the anterior surface of the gastric tube, then the gastric tube was inverted. C, After inversion of the gastric tube. By inverting and retracting the gastric tube (yellow arrow), the working space in the retrosternal cavity was increased, and dissection could be facilitated under the moderate traction, while safely identifying the boundary between the gastric tube and the pleura. D, Findings in the posterior sternum after resection. Left internal mammary artery (yellow arrow) and left lung was exposed, but was not injured after resection of the gastric tube

**FIGURE 3:**
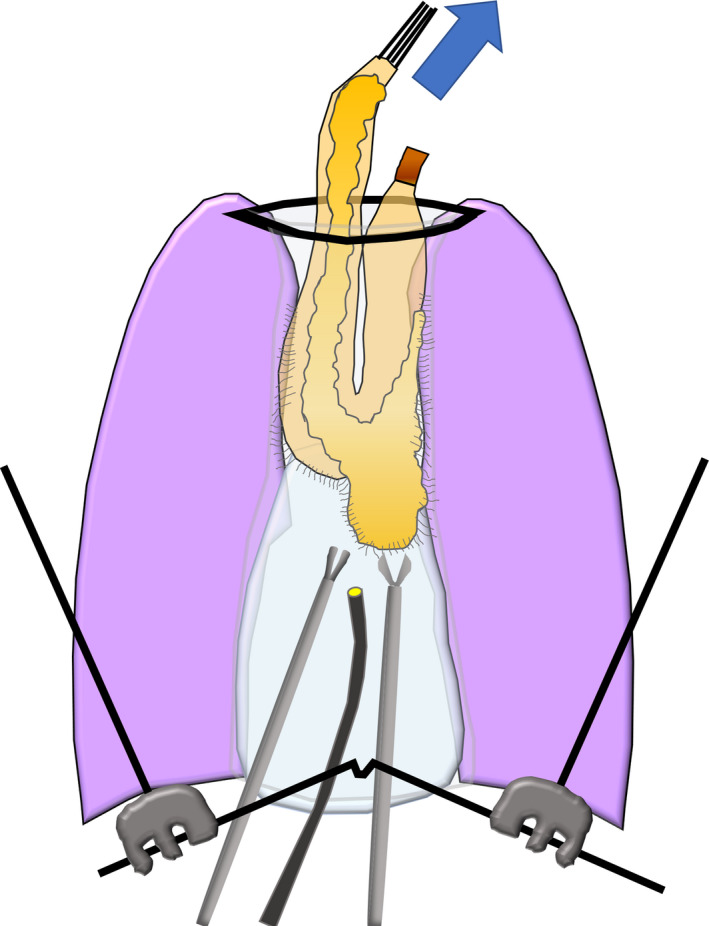
Schema of the gastric tube inversion technique. The silken threads suturing the caudal stump of the gastric tube were guided through the anterior surface of the gastric tube to the cervix and the gastric tube was inverted to create a favorable view of the retrosternal space. By inverting and retracting the gastric tube to the cranial side (Arrow), the working space in the retrosternal cavity was increased and sharp dissection was facilitated under moderate traction, while safely identifying the boundary between the gastric tube and the surrounding organs

Under general anesthesia, the patient was placed in the supine position. An upper abdominal midline incision was performed (Figure 1A) and the adhesions around the gastric tube were divided. Following lymph node dissection around the right gastric and right gastroepiploic arteries, the duodenum was dissected using a linear stapler and the gastric side of the stump was sutured with five silken threads for traction. The open wound in the upper abdomen was retracted with a Kent retractor (Takasago Medical Industry Co. Ltd.) to secure the operative field (Figure 1B). At the entry hole to the retrosternal space, sharp dissection was performed under direct vision between the lower part of the gastric tube and the diaphragm. A collar‐shaped skin incision was simultaneously made in the cervix (Figure 1A), and the cervical esophagus was identified and separated from the surrounding tissues, then the cervical esophagus was taped. We used a 5‐mm flexible scope and performed the gastric tube resection under laparoscopic guidance from the caudal side entry to the retrosternal space. Based on our experience, posterior adhesions were relatively loose in the retrosternal space and could be bluntly dissected (Figure 2A). To facilitate securing the posterior side of the operative space, we first dissected the gastric tube adherent to the anterior aspect of the gastric tube to the posterior sternum. Next, the anterior aspect of the gastric tube was dissected up to the cervical wound. Because the stapled line of the gastric tube was tightly adhered to the surrounding organs, special care was taken during sharp dissection of the adhered side. When the mediastinal pleura was opened during the previous esophagectomy, adhesions to the lung developed, making the dissection extremely difficult. As the dissection advanced toward the cephalad side, the operative space narrowed and the surgical procedures became more difficult. Next, the threads suturing the caudal stump of the gastric tube were guided through the anterior surface of the gastric tube to the cervix (Figure 2B) and the gastric tube was inverted to create a favorable view of the retrosternal space (Figure 3). By inverting and retracting the gastric tube, the working space in the retrosternal cavity was increased and sharp dissection was facilitated under moderate traction, while safely identifying the boundary between the gastric tube and the surrounding organs, including the brachiocephalic vein (BCV), internal mammary vessels (IMVs), and/or pleura (Figure 2C). After the gastric tube was completely dissected (Figure 2D), the surgical specimen was extirpated from the cervix and the cervical esophagus was divided. Bowel reconstruction was routinely restored by the jejunum via the subcutaneous route. However, colon reconstruction via the retrosternal route was performed when the patient did not desire the bowel reconstruction by the subcutaneous route.

## 3 RESULTS

The patients’ clinical characteristics are summarized in Table 1. All of the patients were males. Poorly differentiated adenocarcinoma was the most common pathologic diagnosis. Total gastric tube resections were completed using a laparoscopic mediastinal approach without a median sternotomy in all cases. With respect to intraoperative complications, a pulmonary injury occurred in one case and was repaired using TachoSil^®^ (CSL Behring KK Japan). No major vessel injuries occurred. The operative outcomes and postoperative complications are summarized in Table 2. The median laparoscopic operative time was 130.5 min and the median total operative time was 543.5 min. Of the six patients, one who was diagnosed with type 4 gastric cancer resulted in a microscopical residual tumor resection, whereas the remaining five patients underwent curative resections. Clavien‐Dindo (CD) classification more than a grade 3 postoperative complication occurred in two of six cases (33.3%). Of the two cases, one was a superficial surgical site infection of the cervix. The other case involved a prostate abscess arising from a urinary tract infection; transurethral drainage of the abscess was performed under general anesthesia. Postoperative pneumonia (CD grade 2) occurred in two cases. The median length of postoperative hospital stay was 29.5 days (range, 16‐60 days), and no in‐hospital deaths occurred.

**TABLE 1:** Baseline clinical characteristics of patients with total gastric tube resection undergoing laparoscopic transretrosternal approach

Characteristics	Total
	*n* = 6
Age, years (median[range])	68.5 [ 58‐78 ]
Gender	
Male/Female	6/0
Main tumor location	
U/M/L	1/2/3
Clinical tumor invasion depth	
cT1/cT2/cT3/cT4	3/2/1/0
Clinical lymph node metastasis	
cN0/cN1/cN2/cN3	5/0/1/0
Histology	
tub/por/sig	1/3/2
Pathologic tumor invasion depth	
pT1/pT2/pT3/pT4a	3/2/0/1
Pathologic lymph node metastasis	
pN0/pN1/pN2/pN3	4/0/1/1

Note

Tumor location was classified according to the 15th edition of the Japanese Classification of Gastric Carcinoma.

Abbreviations: Por, poorly differenciated adenocarcinoma; sig, signet ring cell carcinoma; tub, tubular adenocarcinoma.

**TABLE 2:** Surgical outcomes and postoperative complications

Variables	Total
	*n* = 6
Operative measures	
Total operation time, min, median [range]	543.5 [ 429‐686]
Laparoscopic operation time, min, median [range]	130.5 [ 83‐145]
intraoperative blood loss, ml, median [range]	250 [170‐350]
Residual tumor (R0/R1/R2)	5/1/0
Postoperative complications	
C‐D grade ≧3, n (%)	2 (33.3%)
Pneumonia	2 (33.3%)
Anastomotic leakage	0 (0%)
Recurrent nerve palsy	1 (16.7%)
Superficial SSI	1 (16.7%)
Lymphorrhea	0 (0%)
Reconstructed organ necrosis	0 (0%)
Delirium	1 (16.7%)
Urinary infection	1 (16.7%)

Abbreviations: C‐D, Clavien‐Dindo classification, SSI: Surgical site infection.

## 4 DISCUSSION

This novel gastric tube inversion technique combined with a laparoscopic mediastinal approach was shown to be safe and less invasive in gastric tube resection for gastric tube cancer reconstructed through the retrosternal route, thereby avoiding the risk of severe complications associated with a median sternotomy. Moreover, the gastric tube inversion technique helped secure a sufficient working space in a narrow operative field and allowed for safe dissection with a favorable surgical view.

Some authors have reported a radical surgical procedure for gastric tube cancer reconstructed through the retrosternal route without a sternotomy.^7^, ^8^, ^9^, ^10^ The approaches described in these reports were mainly classified as laparoscopic mediastinal and thoracoscopic approaches.

The usefulness of the laparoscopic mediastinal approach has been reported, but all authors acknowledge a disadvantage of this approach is the narrow surgical field, especially in the upper mediastinum^7^. Furthermore, large blood vessels, including the superior vena cava (SVC), BCV, and IMVs are present around the gastric tube in the upper mediastinum, and special care must be taken when performing the dissection between the large vessels and the gastric tube. Various innovations have been reported to secure the working space in the upper mediastinum. Hosoya et al^9^ lifted the anterior chest with two Kirschner wires inserted subcutaneously and an elevated handle; however, this procedure requires special instruments and there is limited versatility. Shiozaki et al^10^ described a procedure which uses the clasps of a Kent retractor with hooking onto not the caudal and cranial edges of the sternum. Although both techniques can slightly increase the upper mediastinal space of the retrosternal cavity, these techniques do not have the potential to significantly improve the narrow surgical field. To facilitate dissection in the upper aspect of the mediastinum, Kimura et al^7^ reported the effectiveness of an additional intercostal port in performing the laparoscopic mediastinal approach. Kimura et al^7^ demonstrated that retraction of the gastric tube can be improved by inserting forceps from an additional intercostal port. Although an additional intercostal port may improve retraction of the gastric tube, this may be less useful for widening the upper mediastinal surgical field. In contrast, using our gastric tube inversion technique can provide a favorable surgical field in the upper mediastinum, thus dissection between the gastric tube and the surrounding organs can be performed with good operative view.

Recently, a trans‐mediastinal radical esophagectomy has been reported.^11^, ^12^ Fujiwara et al^11^ concluded that a single‐port mediastinoscopy is safe and a meticulous mediastinal lymphadenectomy can be performed under favorable expansion of the mediastinal space by carbon dioxide insufflation or a forced pneumomediastinum. Furthermore, a Japanese report^13^ showed that the laparoscopic mediastinal approach can be performed safely under favorable expansion of the retrosternal space by carbon dioxide insufflation during resection of the retrosternal gastric tube. Indeed, the gastric tube had been reconstructed by open approach in the other five cases, but only in the sixth case, the gastric tube had been reconstructed by laparoscopic approach. Therefore, we initially started the sixth case using a laparoscopic approach because we expected that there would be less intra‐abdominal adhesions and that the pneumomediastinum would provide a better view of the mediastinum. However, we converted the laparoscopic mediastinal approach to a laparotomy because of limited forceps use in the protruding area of the posterior sternum. Depending on port placement and the size of the retrosternal space, it may be possible to perform retrosternal gastric tube resection using a laparoscopic mediastinal approach under carbon dioxide insufflation, which may help improve the working space with insufflation pressure.

A thoracoscopic approach is also attractive with respect to forceps accessibility. Horie et al^8^ reported a left thoracoscopic approach for resection of the reconstructed gastric tube through a retrosternal route. This approach provides a good surgical view in cases with few adhesions in the left thoracic cavity. Moreover, there are few restrictions on the operative angle for the forceps; however, preoperative evaluation of intrathoracic adhesions is difficult, thus the indication for this approach may be limited. Indeed, the dissection between the SVC or BCV and the gastric tube requires special attention due to the narrow field of view, even with the left thoracoscopic approach.

Although the IMVs course along the posterolateral side of the sternum, there are no vessels in the midline of the posterior sternum. Therefore, we can safely dissect the anterior aspect of the gastric tube to the cervix. By connecting the dissection to the cervix, we can guide the silken threads to the cervix and perform the gastric tube inversion. When the staple line of the gastric tube is on the anterior aspect, dense adhesions often exist between the gastric tube and the posterior aspect of the sternum. In such cases, it is necessary to perform sharp dissection of the adhesions laparoscopically and it is essential to keep the midline of the sternum intact for the prevention of vascular injury during the dissection. In addition, it is also advisable to perform a full circumferential dissection of the lower half of the gastric tube before inverting the gastric tube. These preparations make it possible to invert the gastric tube. We were able to perform the gastric tube inversion technique in all six cases. Furthermore, we performed sharp dissection with moderate traction under a good surgical view by pulling the inverted gastric tube from the cervical side. As mentioned above, using the laparoscopic mediastinal approach, securing the surgical view and operability in the upper mediastinum can be problematic. Therefore, we believe that our novel gastric tube inversion technique, which can overcome these shortcomings, is useful and safe. This method may not be suitable for large tumors in which the gastric tube cannot be inverted, but in such cases a total gastric tube resection with a median sternotomy may be indicated. Another limitation of this technique is that it is only available when gastric tube cancer is reconstructed by the retrosternal route.

In conclusion, the novel gastric tube inversion technique combined with a laparoscopic mediastinal approach is safe and effective and may become a standardized surgical procedure for gastric tube cancer reconstructed through the retrosternal route.

## DISCLOSURE

Funding: Authors have no financial ties to disclose.

Conflict of interest: The authors declare that they have no conflicts of interest.

Author Contribution: Tetsuya Abe participate in treating the patients, searching for literature, drafing the manuscript, and making the tables and figures. Yoshihisa Numata participated in treating the patients and helped to analyze the data. Eiji Higaki and Takahiro Hosoi participated in treating the patients. Yasuhiro Shimizu participated in planning the treatments. All authors read and approved the final manuscript.
